# Botanical Report

**Published:** 1813-05

**Authors:** 


### BOTANICAL REPORT.

THE eleventh volume of that highly interesting work, THE ASIATIC RESEARCHES, published in Calcutta, has been lately reprinted in London. Among many other valuable materials, this volume contains an interesting botanical paper by Dr. Roxburgh, entitled "Descriptions of several of the Monandrous Plants of India, belonging to the Natural Order called *Scitamineæ* by Linnæus, *Cannæ* by Jussieu, and *Drimyrhizæ* by Ventenat." The first from the Latin word *Scitamentum*, because from this natural order many of the condiments, with which our food is wont to be flavored, are derived, such as ginger, turmerick, &c.; the second from a chief plant in the family, according to the principles of Jussieu; the last from the general prevailing warmth or biting quality in the roots of these plants.

Nearly the whole of the plants of this natural order are natives of a tropical climate; and the nature of their flowers not admitting of preservation, by drying, in a state very fit for the examination of the organs of fructification, it is not to be wondered at that they should have remained, for a long period, but little known to European botanists; more especially as they require to be cultivated in the bark-stove in our northern latitude, and even with this artificial assistance are more difficult to bring into flower than some other orders. Kœnig was the first of the pupils of Linnæus that resided long enough in India to acquire a considerable knowledge of this family. He described many of them with great accuracy, but his herbarium unfortunately perished, and, as he did not live to publish any account of them himself, the botanical world are only acquainted with his labors through the writings of A. I. Retzius, professor of natural history at Lunden, in Sweden, except such as may have had an opportunity of consulting his manuscripts preserved in the Banksian library.

Retzius, however, from Kœnig's descriptions alone, characterised the genera better than had been done before, and his arrangement has been almost implicitly followed by Willdenow, in his new edition of the *Species Plantarum.* He has likewise the particular merit of first pointing out the importance of the *anthera,* or more properly of the *stamen,* in distinguishing the genera, which has been since pursued with so much success by Roscoe, in his account of this natural family, published in the eighth volume of the Transactions of the Linnæan Society, whose arrangement has been adopted by Mr. Dryander, in the new edition of the Hortus Kewensis.

Of the labors of Roscoe, Dr. Roxburgh has made ample use; his genera are almost the same, but having had a much better opportunity of examining living plants from his favorable situation, as prefect of the botanic garden at Calcutta, he has of course described many species unknown to Mr. Roscoe. He has only ten genera, and of these Canna and Phrynium are not true *Scitamineæ;* of the former he makes only one species, of which both the yellow and red varieties are common throughout India, but differ in no respect in the form of the flower; and he therefore presumes that the C. *lutea* of the Hortus Kewensis must be different from his yellow variety.

Phrynium contains three species: 1. *Dichotomum,* (Thalia *canneformis,* Linn.) Of the split stems of this plant mats are made, esteemed for their smoothness and cool feel. 2. *Virgatum,* from Dr. Anderson's garden at Madras. 3 *Capitatum,* of which a figure is given. Thalia *geniculata* and *dealbata* not being natives of the East Indies are known to Dr. Roxburgh only through descriptions. They do not belong to the same genus.

Of Hedychium he has only one species, the *coronarium* of Linnæus, figured in the Botanical Magazine, No. 708.

Of Kœmpferia he has four species, viz. *Galanga, rotunda, angustifolia,* and *pandurata;* of these the two former are both figured in the Botanical Magazine, although these figures are not quoted by the author, as he has usually done; the two latter are new, and of the last the author has presented us with a figure.

Of Curcuma he describes fourteen species. As the economy of the roots of these plants are very curious, we shall transcribe Dr. Roxburgh's account of them. "In this family," says the doctor, "as well as in the other herbaceous genera, the root is biennial, and consists of what I shall call *Bulbs, Tubers,* and *Fibres.* The former are, during the first year, like other bulbs, formed in the centre of the bases of the sheaths of the leaves; and may, during this period, be

4 called called phyllophorous (leaf bearing) receptacles. These bulbs have, on their opposite sides, a vertical row of *buds*, corresponding with the number of the bifarious leaves, and sheaths, (there being one in the axil of each) which grew on the bulb. From these buds, or eyes, issue the *palmate tubers*," [as these are totally different from what Dr. R. afterwards calls the *tubers*, he should have called them by a different name, as *rhizomata*, or *rootstocks*,] "which proceed nearly horizontal, in opposite directions, and branch out, more or less, according to the nature of the plant, &c. From the lower part of the bulbs the *fibres* or general roots chiefly spring; these are strong, thick, long fibres, with numerous small fibrils from their sides, penetrate deep into the soil, in various directions, and in by far the greater number of species, if not in all, several of them terminate in a single oblong *tuber*. These are invariably less deeply colored, and less fragrant than the ovate bulbs and palmate tubers of the same plant. In various parts of India the natives prepare from these tubers, and from no other part, a very fine pure white *starch* or *fecula*, which they use medicinally, and as an article of diet. It is every way like what is met with under the name *arrow-root*; and the process for obtaining it, exactly as described by Dr. Wright, for obtaining that substance from the roots of Maranta *arundinacea*. That followed by the Malays is mentioned by Rumphius, in his Herbarium Amboinense."

The species of CURCUMA are, 1. *Zedoaria*, (Amomum *Zedoaria*, L.) 2. *Zerumbet*, (Amom. *Zerumbet*, Lin.) The roots of this species are said to be determined to be the true Zedoary of the London druggists. 3. *Cæsia*. 4. *Æruginosa*. 5. *Ferruginea*. 6. *Rubescens*. 7. *Comosa*. 8. *Leucorhiza*. 9. *Angustifolia*. The seven last mentioned species are all new. To the last, of which we are presented with a figure, there is added a note from the pen of the president, Thomas Colebrooke, esq. chief judge of the supreme civil and criminal courts for the natives of Bengal, upon the subject of the nutritious powder prepared from the tubers of these plants. This note we shall transcribe for the information of some of our readers.

"From the tubers of the roots of this plant (Curcuma *angustifolia*), and of C. *leucorhiza*, Tikhur, a sort of starch or flour, like *arrow-root*, is prepared, by a very simple process. The *Kherwars*, one of the tribes of mountaineers inhabiting the forests of the Vind'hya mountains, use the following method, according to the information which I received when traversing those forests. The roots are ground, and water is added in considerable quantity. The starch or flour settles at the bottom of the vessel; and, the water being then poured off, the starch is dried in the open air. From eight parts by weight of the root, one part of starch or flour is obtained. It is said to be commonly bartered by the Kherwars south of the Sone for an equal weight of salt.

"Having reason to believe that the same sort of starch or flour is also obtained in the district of Chatgaon, I applied to Mr. Macrae, surgeon at that station, and received very satisfactory information. The powder obtained at Chatgaon from the roots is well known by the name of *Tikhur*, and the plant itself is there called Phalepa and Cachûr. Judging from the specimens of the leaves and roots, which were were received from Mr. Macrae, I have little doubt that the plant is allied to this species, and probably belongs to the kindred one of C. *leucorhiza*. The powder, prepared from the root, is considered by the natives at Chatgaon as an excellent restorative in cases of consumption; and a preparation of it, in the form of a sweetmeat, is sold in the market.

"I shall only add, on the subject of this nutritious powder, that it is very similar to the powder obtained in America from the roots of Maranta *arundinacea*, and which is known in Europe by the name of Indian arrow-root; and there is reason to believe that other plants of the same natural order afford a similar produce.

"In regard to the Asiatic names of the plant, and of its produce, I am unable to add any well-ascertained synonyma to the received name of *Tikhur*. It is unnoticed under this denomination in the works of Hindu and Muhammedan writers on the materia medica of India; and the name of Cachûr, by which the plant is distinguished in Chatgaon, properly belongs to the Zerumbet (Curcuma, Z.)"

The above nine species of Curcuma bear their spikes of flowers laterally; the remainder belong to another section, bearing their flowering spikes in the centre. 10. C. *longa*, Lin. The rootstocks of this plant are the Turmeric of the shops. 11. C. *Amada*. The root of this plant is an ingredient in the Indian curries. 12. C. *viridiflora*. 13. C. *montana*. Coromand. plants, 2. No. 151. 14. C. *reclinata*. Besides the above species there are others in the botanic garden at Calcutta, which have not yet flowered, making in the whole about twenty species of this interesting genus.

Of Amomum, as now constituted, the species are very limited; four only are enumerated by Dr. Roxburgh, viz. 1. A. *Cardamomum*. The Cardamomum minus of Rumf. but a very different plant from that which bears the true lesser Cardamoms of our shops, but the seeds being agreeably aromatic, are used by the Malays for the same purposes. 2. *Angustifolium*. 3. *Aculeatum*. Of this species a figure is given. 4. *Maximum*. In the new edition of the Hortus Kewensis there is only one species of Amomum; another is described by Dr. Smith in Exotic Botany; both are natives of Africa, and quite distinct from the four here described; it may be doubted even if they really belong to the same genus.

The genus Zingiber, separated by Mr. Roscoe from that of Amomum, is more numerous; nine species are here described, of which the two last having terminal (caulescent) not radical spikes, may probably be distinct. 1. Zingiber *officinale;* the plant which produces the true ginger. 2. *Zerumbet*. The figure of this plant in Exotic Botany, tab. 112, is not here quoted. This plant, (says the President, in a note,) was supposed by Rumphius to be the Zerumbet; and the Brahmens, who assisted Van Rheede, appear to have taken it for the Galangale. It is neither of these drugs, but bears more resemblance to the next species. 3. *Cassumunar*, which is here figured, and has been given also in the Botanical Magazine. The root of this plant is supposed to be the true Cassumunar, now hardly known in our shops. 4. *Roseum*. Amomum *roseum*. Corom. pl. 2. No. 126.

5. *Ligu-*

5. *Ligulatum.* 6. *Rubens.* 7. *Squarrosum.* 8. *Capitatum.* 9. *Marginatum.* The seven last species all new.

Of COSTUS only one species occurs, the *speciosus* of Dr. Smith and Hortus Kewensis; which was mistaken by Jacquin and others for C. *Arabicus* of Linnæus.

Of ALPINIA Dr. Roxburgh describes eight species. This genus, since Mr. Roscoe's publication, is much better understood than before. But both these authors unite HELLENIA of Linnæus with it; which is again separated by Mr. Brown, and that too on Mr. Roscoe's principles, as having the filament extended beyond the anther; the pericarp too instead of being berried is crustaceous.

The ALPINIAS have rather lofty stems, which are generally perennial, or at least biennial, and bear their flowers in large showy racemes at their extremities. The only exception to this kind of inflorescence is afforded by A. *Cardamomum;* which for this reason ought to have been excluded from the genus. The species, according to our author, are, 1. A. *Galanga.* The roots of this plant were ascertained by Sir Joseph Banks and Dr. Coombe to be the true *Galanga major* of the shops; but which are now very little, if at all, in use. A. *Allughas;* Hellenia *Allughas* of Linnæus. 3. A. *malaccensis,* said by our author to be the most stately and beautiful plant of any in the whole order found in the East Indies. This species has not, we believe, been as yet introduced into this country. 4. A. *nutans,* Exotic Botany, tab. 106. Renealmia *nutans* of the Botanist's Repository, a plant well known in the stoves of the curious, and certainly a very magnificent one. 5. A. *mutica;* a new species. 6. A. *calcarata;* Renealmia *calcarata* of the Botanist's Repository. 7. A. *Cardamomum;* Elettaria *Cardamomum* of Dr. Maton, Transactions of the Linnean Society, vol. 10, p. 254. The doctor does not, however, seem to have been aware that this plant corresponds with the genus Alpinia as defined by Mr. Roscoe, and is hardly to be separated from it, except on account of its very different inflorescence, which is radical, as had been before remarked by Mr. Brown, in his valuable Prodromus. The seeds of this plant are the true lesser cardamoms of the shops. 8. A. *spicata;* a new species, and the smallest in the whole genus.

Of the genus GLOBBA, nothing was understood by botanists till Dr. Smith discovered, from the Linnean Herbarium, what the species, here first mentioned, 1. Globba *marantina,* really was, and gave a figure of it in his Exotic Botany, together with two other species of the same genus found by Dr. Buchanan. Prior to this discovery, Dr. Roxburgh had considered this genus as new, and had named it COLEBROOKIA, in honor of Thomas Colebrook, esq. chief judge of the supreme civil and criminal courts for the natives of Bengal. 2. G. *bulbifera;* probably the same species as *sessiliflora* of Dr. Sims, in Botanical Magazine, No. 1428. 3. G. *orixensis;* a new species, of which a figure is added. 4. G. *Hura;* Hura *surinamensium* of Retzius. 5. G. *pendula;* a new species. 6. G. *radicalis;* the Mantisia *saltatoria* of Dr. Sims; Bot. Mag. No. 1320: separated from Globba, chiefly on account of its inflorescence, as Elettaria from Alpinia, &c. There is one circumstance in which, according to Dr. Roxburgh,

Roxburgh, the last-mentioned genus differs perhaps from all the rest of the scitamineæ, which is, that the capsules are one-celled, and the receptacles parietal; that is, the seeds are attached to the circumference of the capsule, and not at the inner angles of the cells of the capsules, as in all the other genera. By this insertion of the seeds, Globba approaches the natural order of *Orchideæ*.

Upon the whole, we consider this communication by Dr. Roxburgh as being highly interesting, and a very valuable addition to our knowledge of this natural family. We do not, however, altogether approve of the language of this dissertation, which is not unfrequently obscure. We should have been better satisfied if the author had adhered more closely to the lucid diction of Mr. Roscoe; and that, instead of endeavoring to fabricate a system of his own, and which after all differs very little from that of his predecessor, he had compared the characters of the numerous species he describes by the principles of Roscoe, and had pointed out where these were deficient, or otherwise, in establishing the genera. As Mr. Roscoe's system is erected upon the form of the filament, and its mode of supporting the anther; and Dr. Roxburgh makes no mention of the filament at all, it may appear at first sight, that the two systems are materially different; but it is only in appearance, the doctor having merely changed the language, by speaking of the filament as if it were a part of the anther; thus, what Roscoe terms a production of the filament beyond the anther, the other, with less accuracy, considers as a crest of the anther. We do not disapprove of his calling to his assistance, in characterising the genera, the form of the inner border of the corolla, as Mr. Brown had also done before him, in the inestimable work we have before mentioned; a work, which had Dr. Roxburgh studied with the care he ought to have done, before he sat down to write his essay, and had kept the principles there divulged constantly in his view whilst composing his descriptions, we will venture to assert that he would have written a dissertation, which would at the same time have been more creditable to himself, and, in a great degree, more useful in promoting the science of botany.

